# Surface-Anchored Monomeric Agonist pMHCs Alone Trigger TCR with High Sensitivity 

**DOI:** 10.1371/journal.pbio.0060043

**Published:** 2008-02-26

**Authors:** Zhengyu Ma, Kim A Sharp, Paul A Janmey, Terri H Finkel

**Affiliations:** 1 Department of Pediatrics, University of Pennsylvania School of Medicine, Philadelphia, Pennsylvania, United States of America; 2 Department of Biochemistry and Biophysics, University of Pennsylvania School of Medicine, Philadelphia, Pennsylvania, United States of America; 3 Institute for Medicine and Engineering, University of Pennsylvania School of Medicine, Philadelphia, Pennsylvania, United States of America; 4 The Children's Hospital of Philadelphia, Philadelphia, Pennsylvania, United States of America; National Jewish Medical and Research Center, United States of America

## Abstract

At the interface between T cell and antigen-presenting cell (APC), peptide antigen presented by MHC (pMHC) binds to the T cell receptor (TCR) and initiates signaling. The mechanism of TCR signal initiation, or triggering, remains unclear. An interesting aspect of this puzzle is that although soluble agonist pMHCs cannot trigger TCR even at high concentrations, the same ligands trigger TCR very efficiently on the surface of APCs. Here, using lipid bilayers or plastic-based artificial APCs with defined components, we identify the critical APC-associated factors that confer agonist pMHCs with such potency. We found that CD4^+^ T cells are triggered by very low numbers of monomeric agonist pMHCs anchored on fluid lipid bilayers or fixed plastic surfaces, in the absence of any other APC surface molecules. Importantly, on bilayers, plastic surfaces, or real APCs, endogenous pMHCs did not enhance TCR triggering. TCR triggering, however, critically depended upon the adhesiveness of the surface and an intact T cell actin cytoskeleton. Based on these observations, we propose the receptor deformation model of TCR triggering to explain the remarkable sensitivity and specificity of TCR triggering.

## Author Summary

Using the T cell receptor (TCR) as a sensor, T cells of the immune system constantly migrate in lymphoid organs and probe the surface of antigen-presenting cells (APCs) for foreign antigens, a sign of pathogen infection. Antigen binding by TCRs leads to T cell activation and subsequent immune response to combat the pathogens. Interestingly, although T cells respond well to antigens on APCs, they do not recognize the same antigens in solution. What is it that makes antigens on APCs recognizable? To address this, we used lipid bilayers and plastic surfaces to construct artificial APCs with defined antigen number, composition, and configuration. We found that T cells respond to very few individual foreign antigens on artificial APCs, and contrary to some current opinion, formation of antigen clusters on APCs is not required for antigen recognition by T cells. TCR triggering, however, requires T cell adhesion to the APC surface and then occurs only if the T cells are able to move. We propose that at the dynamic T cell–APC interface, antigen on APCs activates T cells by applying force to the TCR and deforming its structure, which cannot be achieved by soluble antigens due to their lack of anchorage.

## Abstract 3

Why is it that T cells are blind to antigens in solution but highly sensitive to antigens anchored on a surface? The authors show that this is not due to antigen clustering, but could involve mechanical forces associated with cell locomotion.

## Introduction

Using T cell receptors (TCRs) as sensors, T cells probe the surface of antigen-presenting cells (APCs) for the presence of antigenic (agonist) peptides presented by major histocompatibility complex (pMHC) molecules. Engagement of TCRs by agonist pMHCs initiates a signal, which is transmitted to the nucleus via kinase cascades and protein translocation, leading, ultimately, to T cell activation. In clear contrast to advances in our understanding of intracellular TCR signaling pathways, the question of how binding of pMHC initiates or triggers TCR signaling remains unclear [1]. This is despite knowledge gained from the study of triggering mechanisms of other receptor systems, for example, the conformational change of G protein-coupled receptors and the dimerization of growth factor receptors. The difficulty in resolving the mechanism of TCR triggering is attributed to the complexity of multichain TCR/CD3 structure, the diversity of peptides presented by MHCs on APCs, and importantly, the complex environment where pMHC-TCR interaction takes place.

One fundamental question still unanswered is the minimum requirements for TCR triggering. In terms of the pMHC ligand, a critical question is whether a monomeric agonist pMHC alone can trigger TCR independently, or whether it must act cooperatively with another neighboring pMHC, either agonist or endogenous, as a dimer to simultaneously engage, and in effect crosslink, two TCRs. In terms of the environment or the context of TCR triggering, critical questions are whether soluble agonist pMHCs are capable of triggering TCR or if they must be surface-anchored, whether molecules other than pMHCs on APCs, e.g., costimulatory molecules, contribute to TCR triggering, and whether and how physical and mechanical aspects of the T cell–APC interaction play a role in TCR triggering. There are reports showing that TCR is no different from receptors for soluble ligands, e.g., hormone receptors, and that soluble monomeric pMHCs are sufficient to trigger TCR [2–4]. These data suggested that TCR must be triggered through either TCR conformational change or heterodimerization of TCR and CD4 or CD8 coreceptor via simultaneous pMHC binding. In these studies, however, binding of peptides [5] or pMHC molecules to the surface of the cell or culture dish was difficult to rule out. Mechanistically, with one exception [6], rather extensive crystallography studies have not revealed global large-scale conformational changes of TCR after engagement of agonistic pMHCs [7–14]. It is also uncertain whether coreceptors play a role in TCR triggering, since TCR triggering in vitro and in vivo [15,16] can occur in the complete absence of CD4 or CD8. Moreover, these studies are contradicted by data showing the total lack of TCR triggering capability of soluble agonist pMHCs in solution even at very high concentrations [17–22]. In sharp contrast, however, many studies have demonstrated the remarkable potency of a few agonist pMHCs on APCs to trigger TCR [23–25]. If TCR triggering by agonist pMHCs depends upon the context of the APC, then the question becomes what else at the interface of T cell–APC interaction contributes to TCR triggering.

Based upon observations of T cell activation by antibody crosslinking, TCR crosslinking by agonist–agonist pMHCs has been proposed as the mechanism of TCR triggering. This theory relies upon the presence of putative agonist–agonist pMHCs on the APC surface, and is supported by observations that a significant fraction of MHCs on APCs are immobile [26,27]; thus, two agonist pMHC monomers could act as a dimer if they are immobilized close to each other. However, since TCR can be triggered by only a few agonist pMHCs amongst a huge number of endogenous pMHCs on the APC, the chance of agonist–agonist pMHC formation should be very small. Getting around this issue is a recent theory that TCR is triggered by a “pseudodimer” of an agonist pMHC and an endogenous pMHC [28,29], based upon data showing that some endogenous pMHCs enhance TCR triggering by agonist pMHCs. In addition to the possible contribution of endogenous pMHCs on APCs, van der Merwe and colleagues have postulated that the two-dimensional (2D) nature of the pMHC–TCR interaction plays a critical role in triggering. The proposed kinetic-segregation model [30–32] suggests that TCR signal initiation is induced by segregation of small kinase-associating molecules and large phosphatase-associating molecules at the tight junction between a T cell and an APC, leading to a net increase in tyrosine kinase activity proximate to the TCR/CD3 complex. This model would obviate the need for receptor crosslinking and require only monomeric agonist pMHC.

With conflicting data and the multiple models proposed, it is clear that there is no consensus regarding the mechanism of TCR triggering. A solution to this puzzle would be aided by definition of the critical factors involved in the T cell–APC interaction that directly contribute to TCR triggering by agonist pMHCs. Here, we test TCR triggering using artificial APCs consisting of fluid lipid bilayers or fixed plastic surfaces with defined components. We demonstrate that TCR can be triggered by a very low number (1–10) of monomeric agonist pMHCs. TCR triggering is independent of endogenous pMHCs and of any other molecule found on real APC surfaces, but is critically dependent upon (1) surface-anchoring of pMHC, (2) T cell adhesion to the surface, and (3) intact actin cytoskeletal function. Based upon these data, and incorporating the impact of mechanical stress on pMHC-TCR binding kinetics, we propose the receptor deformation model of TCR triggering by monomeric pMHCs on a surface. In this model, TCR is triggered by TCR/CD3 conformational changes induced by a cytoskeletal pulling force, transferred via specific pMHC-TCR interactions with sufficient resistance to rupture under force.

## Results

### Establishment of a Lipid Bilayer-Based Artificial APC System

**Figure 1:**
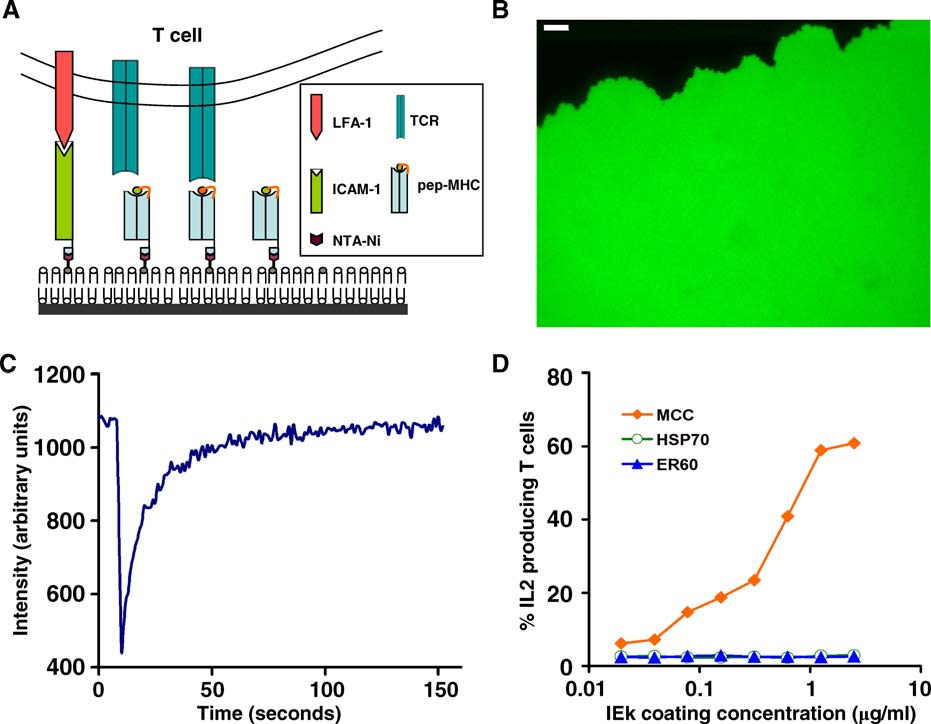
Characterization of the Protein and Lipid Bilayer Components of Artificial APCs (A) Schematic of the interaction between the T cell and the NTA-Ni lipid bilayer-based artificial APC. (B) The expanded area of the POPC bilayer doped with 0.2 mol% fluorescent DOPE-NBD (green). The scale bar represents 10 μm. (C) Fluorescence recovery of a photobleached area (*r* = 4 μm) on the POPC/1mol% DOPE-NBD bilayer. (D) Immobilized agonist, but not endogenous pMHC, stimulates AD10 T cells to produce IL2. The 96-well ELISA plates were coated with IEk proteins at the indicated concentrations. AD10 CD4^+^ T cells were assayed for IL2 production.

To anchor pMHCs on a fluid surface, we developed a planar lipid bilayer-based artificial APC (Figure 1A). This consisted of a glass-supported lipid bilayer anchored with pMHC and ICAM-1, via interactions between nitrilo triacetic acid-Ni (NTA-Ni) lipid head groups on the bilayer and 6× histidine (HisTag) at the membrane-proximal ends of the proteins. An initial consideration was that artificial pMHC dimers may be formed by monomers immobilized in close proximity on lipid bilayers with defects in fluidity or homogeneity. Avoidance of such de facto dimer formation was crucial for interpretation of our experimental data. To this end, a novel technique of lipid bilayer expansion was employed to achieve a higher level of large-scale lipid bilayer homogeneity and fluidity compared to previously reported methods. The newly expanded area of lipid bilayer showed excellent homogeneity and stability in buffer containing bovine serum albumin (BSA), with no defects visible by fluorescence microscopy (Figure 1B). As expected, given its genesis by lipid translocation, the newly expanded lipid bilayer showed a high level of fluorescence recovery after photobleaching (FRAP) of a spot of 4-μm radius, indicating a mobile fraction of close to 100% and a calculated diffusion coefficient of approximately 0.8 μm^2^/sec (Figure 1C), in agreement with typical lipid diffusion rates in biological membranes [33]. The lipids diffuse freely over long range as shown in the recovery of a larger bleaching spot (Figure S1A). Only this newly expanded area of lipid bilayer was used for interaction with T cells. To anchor monomeric pMHC on the bilayer, a lipid with an NTA head group (DOGS-NTA) was doped in the bilayer at 5 mol% and charged with Ni^2+^ to allow binding of soluble ligands with HisTags.

To generate soluble monomeric pMHCs, extracellular domains of MHC class II IEk molecules, each with one of three different covalently linked peptides, were expressed as secreted forms using a baculovirus system in insect cells [34]: IEk-MCC (agonist peptide moth cytochrome c residues 88–103) [35], IEk-HSP70 (endogenous peptide HSP70 234–248) [29,36], and IEk-ER60 (endogenous peptide ER60 448–461) [29,37]. IEk-MCC is a well-characterized ligand for the V_α_11V_β_3 TCR expressed on CD4^+^ T cells from the TCR transgenic mice AD10, AND, and 5C.C7 [38–40]. All IEk proteins were engineered to have an AviTag sequence followed by a HisTag sequence connected to the membrane-proximal ends of the β chain via flexible linkers. The AviTag sequence allows biotinylation of a single lysine residue by the BirA enzyme. All proteins displayed the same single peak corresponding to approximately 50 kDa in gel filtration chromatography (Figure S2A). Their secondary structures were confirmed by circular dichroism spectra (Figure S2B) and thermal melting profiles (Figure S2C), both of which are consistent with that of IEk with bound MCC peptide, described previously [41]. In particular, all three proteins showed similar thermal melting profiles with sharp transitions at high temperature (*T*_m_ = ∼72 °C), indicating that the protein structures are stabilized by bound peptides, since empty IEk without peptide melts at a much lower temperature and with a broad transition [41]. As expected, when immobilized on ELISA plates, only agonist IEk-MCC induced interleukin 2 (IL2) production by primed AD10 T cells (Figure 1D). Moreover, incubation of the endogenous peptide-IEk proteins (IEk-ER60 or IEk-HSP70) with MCC peptides under conditions reported to load 80% of empty IEks [42] did not endow them with T cell activation capacity (Figure S2D). These data indicate that all three IEk proteins were properly folded with their peptide-binding grooves occupied.

Affinity purified IEk proteins were monomers as shown by gel filtration chromatography (Figure S2A), and did not trigger TCR in solution (unpublished data), consistent with previous reports [17–22]. IEk-MCC proteins were never subjected to freezing and thawing, and were always further purified by gel filtration immediately before use. When anchored on the lipid bilayer through HisTag-Ni-NTA binding, IEk proteins moved freely with a diffusion rate only slightly slower than that of the lipids (Figure S1B). The density of IEk on the bilayer was measured at about 2,900 per μm^2^. GFP-HisTag and ICAM-1-HisTag were also expressed in insect cells for use as a control and in enhancement of T cell adhesion, respectively. Thus, we had in hand a lipid bilayer-based artificial APC system, consisting of a lipid bilayer and proteins with the requisite qualities for study of T cell/APC interactions.

### Fewer than Ten Monomeric Agonist pMHCs Are Sufficient to Trigger TCR

**Figure 2:**
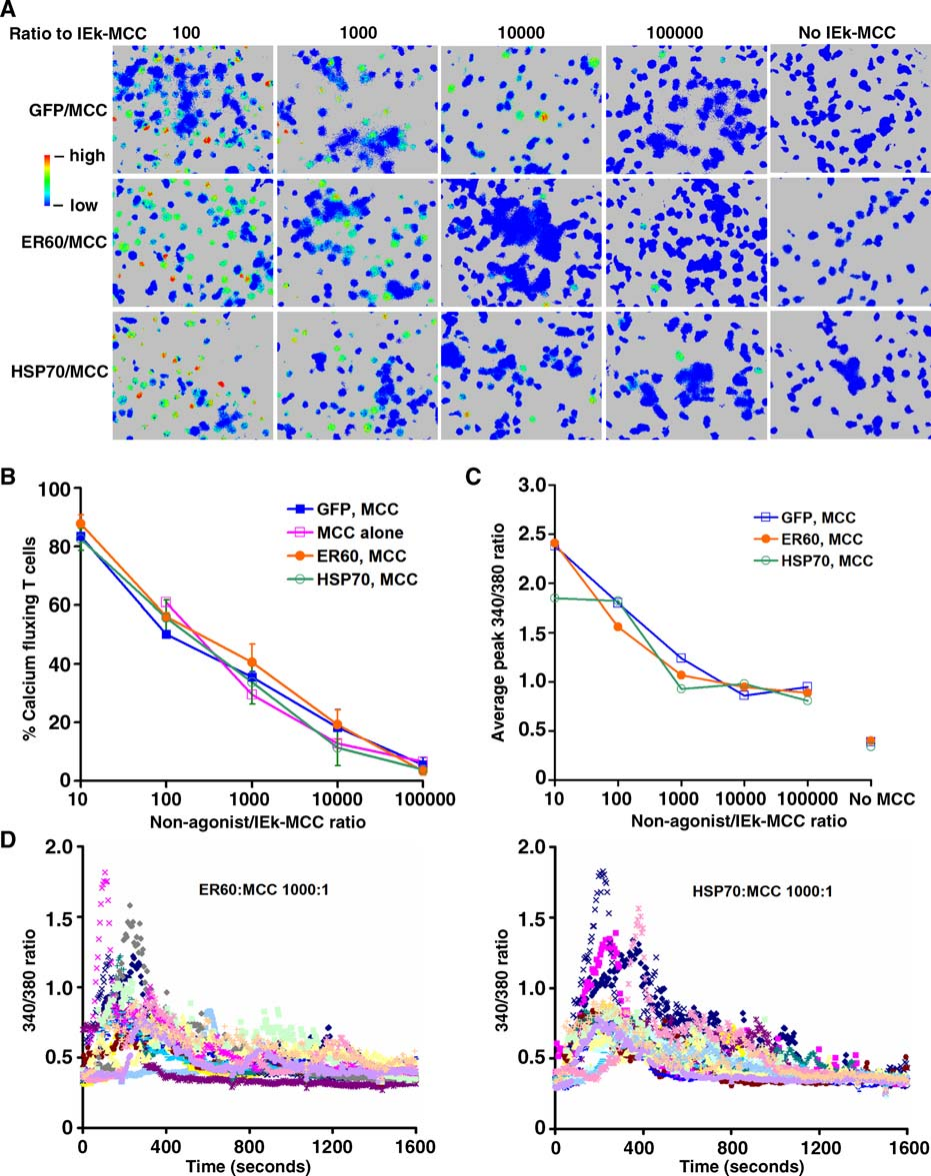
Very Low Numbers of Monomeric Agonist IEk Anchored on a Lipid Bilayer Are Sufficient to Trigger T Cell Calcium Flux, Independent of Endogenous IEk (A) IEk-MCC mixed with GFP-HisTag, IEk-ER60, or IEk-HSP70 at the indicated ratios was anchored on a lipid bilayer containing 5 mol% DOGS-NTA-Ni. Increase in the intracellular calcium level in AD10 TCR transgenic CD4^+^ T cells labeled with fura-2 is indicated by an increase of the 340 nm/380 nm ratio in the pseudocolor images. Bilayers with GFP-HisTag or endogenous IEk only, without IEk-MCC, served as controls. (B) Percentage of AD10 T cells with at least two times increased intracellular calcium levels in response to IEk-MCC alone or mixed with GFP-HisTag, IEk-ER60, or IEk-HSP70 on NTA-Ni lipid bilayers at the indicated ratios. (C) Same as (B), except that the average peak calcium level is shown as the 340 nm/380 nm ratio. The 340 nm/380 nm ratios of T cells stimulated with “No MCC” are average ratios of T cells interacting with lipid bilayers anchored with only GFP-HisTag or endogenous IEks. (D) Change in AD10 T cell intracellular calcium levels over time after contacting NTA-Ni bilayers anchored with IEk-MCC mixed with IEk-ER60 or IEk-HSP70 at a 1:1,000 ratio. Data were compiled from 17 AD10 T cells in each group.

To test the ability of agonist pMHCs alone on a fluid surface to trigger TCR, IEk-MCC was mixed with GFP-HisTag at varying ratios prior to anchoring on the lipid bilayer while maintaining a constant concentration of total protein. ICAM-1-HisTag was added at a concentration equal to one-tenth of the total protein concentration. After contacting lipid bilayers with bound IEk-MCC, primed AD10 T cells demonstrated rapid increases in intracellular calcium, as indicated by an increase in the 340 nm/380 nm ratio (Figure 2A and Video S1). Both the percentage of responding T cells and the amplitude of the increase in intracellular calcium were clearly dependent on the dose of agonist IEk-MCC (Figure 2B and 2C). A 340 nm/380 nm ratio two times above background was observed in T cells interacting with a bilayer anchored with IEk at a GFP:IEk-MCC ratio of up to 100,000:1, whereas no increase in 340 nm/380 nm ratio was observed in T cells interacting with bilayers anchored with only GFP (Figure 2A and 2C, and Video S2). On the basis of the measured size of primed mouse CD4^+^ T cells and the density of IEk on the lipid bilayer, we calculated that, on average, three agonist IEk-MCC monomers were sufficient to trigger a low-level calcium response in about 5% of T cells. The same dose-dependent response was observed when diluted IEk-MCC was anchored on the bilayer alone (Figure 2B).

**Figure 3:**
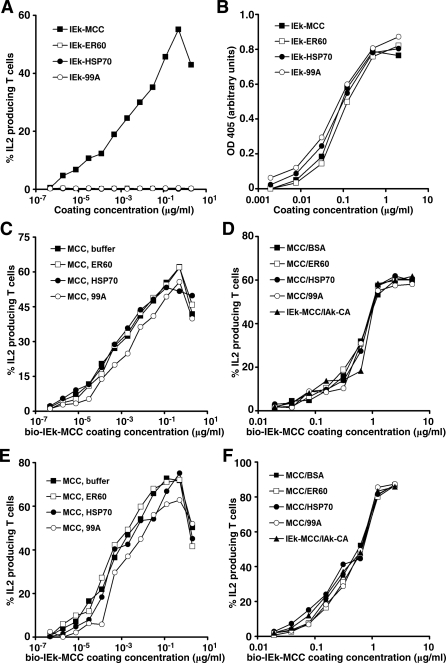
Very Low Numbers of Agonist IEk Anchored on a Fixed Surface Induce AD10 T Cell IL2 Production, Independent of Endogenous or Null IEk, or IAk (A) Bio-IEk-MCC, bio-IEk-ER60, bio-IEk-HSP70, or bio-IEk-99A at the indicated concentrations were added to 96-well plates precoated with streptavidin and incubated for 18 h at 37 °C. AD10 CD4^+^ T cells were added to the wells and stimulated for 7 h prior to IL2 assay. (B) The level of IEk proteins on streptavidin plates coated as in (A) was determined by ELISA using anti-IEk 14-4-4s antibody followed by HRP-conjugated goat anti-mouse antibody. ABTS substrate color development was monitored at 405 nm. (C) Streptavidin plates coated with bio-IEk-MCC at the indicated concentrations were subsequently saturated with 10 μg/ml bio-IEk-ER60, bio-IEk-HSP70, bio-IEk-99A, or buffer for 1 h at room temperature before being used to stimulate AD10 T cells. (D) The 96-well ELISA plates were coated with bio-IEk-MCC at the indicated concentrations overnight at 4 °C. The wells were then saturated with 10 μg/ml BSA, IEk-ER60, IEk-HSP70, IEk-99A, or IAk-CA for 4 h at room temperature. Stimulation of AD10 and measurement of IL2 measurements were done as described in (A). (E and F) Same as (C) and (D), respectively, except that 5C.C7 cells were used.

To test whether TCR triggering is influenced by the fluidity of the pMHC anchoring surface, we immobilized IEk molecules on a fixed plastic surface. IEk molecules biotinylated on the AviTag sequences were diluted and immobilized on 96-well plates precoated with streptavidin for 18 h at 37 °C for thorough binding. Bio-IEk-MCC–coated wells stimulated AD10 T cells to produce IL-2 in a dose-dependent manner (Figure 3A). Approximately 5% of the T cells were activated by a surface coated with bio-IEk-MCC diluted to a concentration of 1.9 × 10^−6^ μg/ml. Assuming that all bio-IEk-MCC molecules bound to the surface of the well, and using 78.5 μm^2^ as the T cell contact area based on the measured diameter of primed T cells, we calculated that 0.83 bio-IEk-MCC molecules per cell, or 83 bio-IEk-MCC per 100 cells, were sufficient for T cell activation. In contrast, surfaces coated with bio-IEk-ER60, bio-IEk-HSP70, or bio-IEk-99A did not activate AD10 T cells even at the highest coating concentration, although they bound comparably to streptavidin-coated wells (Figure 3B). IEk-99A contains a covalently linked MCC peptide with a single K>A mutation at the p5 TCR-interacting position that renders it null for AD10 and 5C.C7 T cells [43,44]. Thus, T cell activation is induced by very low numbers of agonist, but not endogenous or null, pMHC when presented to T cells on either a fluid or a fixed surface.

### TCR Triggering by Agonist pMHCs Is Independent of Endogenous pMHCs

The conventional notion of TCR crosslinking is by two agonist pMHCs. Because of the very low number of agonist IEk needed to trigger TCR and the highly fluid nature of the lipid bilayer, it was unlikely that agonist–agonist IEk dimers were present on the lipid bilayer. It has been reported, however, that endogenous pMHCs dramatically enhance TCR triggering by agonist pMHCs by formation of “pseudodimers” [28,29]. To determine the role of endogenous pMHC in TCR triggering, we replaced GFP-HisTag with IEk-ER60 or IEk-HSP70. AD10 T cells responded similarly to IEk-MCC in the context of IEk-ER60 or IEk-HSP70, as well as to IEk-MCC in the context of GFP (Figure 2A–2C and Videos S1, S3, and S4), both in terms of the percentage of responding T cells and the level of calcium increase. Similar results were found using primed 5C.C7 T cells (unpublished data). In addition, we did not observe any difference between IEk-MCC diluted with IEk-HSP70 or with IEk-ER60 in triggered T cell calcium flux patterns (Figure 2D). This is in contrast to a previous report in which only IEk-ER60, but not IEk-HSP70, worked synergistically with IEk-MCC, reportedly through pseudodimer formation [29].

To facilitate the formation of pseudodimers between IEk-MCC and endogenous IEks, we immobilized bio-IEk-MCC of varying dilutions onto streptavidin-coated 96-well plates. This was followed by incubation with coating buffer, bio-IEk-ER60, bio-IEk-HSP70, or bio-IEk-99A at an experimentally confirmed saturating concentration to occupy remaining free biotin binding sites. We reasoned that binding of bio-IEk-MCC and endogenous pMHC on the same tetravalent streptavidin molecule under these conditions would form stably linked pseudodimers. The distance between pMHCs, but not their orientation, is reportedly important for TCR crosslinking [45], with comparable crosslinking demonstrated for linkers ranging from a direct disulfide bond (0.2 nm) to a peptide crosslinker of 7 nm in length [45]. The distance between two biotin binding sites (∼3 nm) on streptavidin is well within this range [46]. Indeed, streptavidin bound with two or more IEk-MCC is capable of activating T cells in solution [22]. Consistent with our observed calcium responses to IEk on lipid bilayers, saturating the surface with endogenous bio-IEk-ER60, bio-IEk-HSP70, or bio-IEk-99A did not enhance the ability of bio-IEk-MCC to induce IL2 production by AD10 T cells (Figure 3C) or 5C.C7 cells (Figure 3E). These results were confirmed by experiments showing that activation of AD10 T cells (Figure 3D) or 5C.C7 T cells (Figure 3F) by bio-IEk-MCC directly bound to ELISA plates was not enhanced by subsequent saturation binding by IEk-ER60, IEk-HSP70, IEk-99A, or IAk-CA. IAk coexists with IEk on the same APCs in vivo. IAk with covalently linked conalbumin peptide (IAk-CA) activates T cells bearing D10 TCRs (Figure S3). On the ELISA plate, the density of bio-IEk-MCC remained the same with or without subsequent saturation binding by other proteins (Figure S4). The difference in shape and range of the response curves to IEk on streptavidin versus ELISA plates is likely a result of their different surface properties (see Text S1). Therefore, on fluid lipid bilayers or on fixed surfaces, T cell activation induced by agonist IEk-MCC was not enhanced by endogenous IEk, null IEk, or another MHC class II molecule, IAk.

### Skewing Endogenous Peptide Populations on a Real APC Surface Does Not Affect T Cell Activation by Agonist Peptides

**Figure 4:**
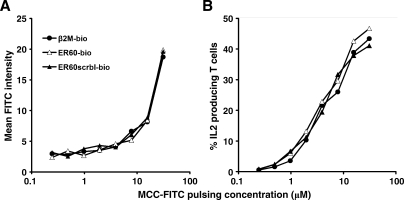
On Real APCs, Endogenous Peptides Do Not Affect T Cell Activation by Agonist Peptides CH27 cells were cultured with 1 mM β2m-bio, ER60-bio, or ER60scrbl-bio peptides for 20 h at 37 °C. After washing, cells were incubated with MCC-FITC peptides at the indicated concentrations for 30 min before washing. (A) The level of MCC-FITC on CH27 cells after pulsing, as measured by flow cytometry. (B) AD10 T cells were mixed with CH27 cells pulsed with MCC-FITC at the indicated concentrations and IL2 production was measured by intracellular staining after 7 h. Data are representative of three independent experiments.

To test the role of endogenous pMHCs in TCR triggering under more physiologic conditions, we skewed the makeup of endogenous peptides on real APCs. We wished to determine whether the ability of APCs to activate T cells could be altered by replacing a major proportion of the original diverse endogenous peptide population with a peptide having putative enhancing or nonenhancing effects on TCR triggering. Murine B-cell lymphoma CH27 cells expressing IEk were incubated with biotinylated β2m peptides (β2m-bio), ER60 peptides (ER60-bio), or scrambled ER60 peptides (ER60scrbl-bio) at high concentrations for 20 h. To determine the degree of IEk occupancy by the biotinylated peptides, pulsed cells were stained with streptavidin-Cy5, and the fluorescence intensity was compared with CH27 cells stained with biotinylated anti-IEk monoclonal antibody, 14-4-4s (14-4-4s-bio), followed by streptavidin-Cy5. The peptides were biotinylated at the N-terminus, so each peptide bound to one streptavidin-Cy5. The number of streptavidin-Cy5 each 14-4-4s-bio antibody could bind was determined using a gel filtration chromatography-based assay (Figure S5), and the result was used to correct for differences in fluorescence intensity. Using this assay, we determined that β2m-bio, ER60-bio, or ER60scrbl-bio peptides occupied about 59%, 17%, or 3%, respectively, of IEk molecules on the surface of CH27 cells (Figure S6). The peptide binding was IEk-specific; only a very low level of binding was observed on the mutant murine B-cell lymphoma, M12.C3, which is deficient for MHC class II (Figure S6). The CH27 cells were then briefly incubated with FITC-labeled MCC peptides (MCC-FITC) before adding to a T cell stimulation assay. MCC-FITC pulsing led to comparable FITC levels on CH27 cells prepulsed with β2m-bio, ER60-bio, or ER60scrbl-bio (Figure 4A), while maintaining relatively consistent IEk occupancy by biotinylated endogenous peptides (unpublished data). In a previous report, only ER60, but not β2m, enhanced T cell activation, presumably by forming pseudodimers with agonist pMHC [29]. If this were the case, then skewing the endogenous peptide population on the CH27 surface towards ER60 or β2m should significantly enhance or reduce, respectively, the chance of pseudodimer formation, and lead to different degrees of T cell activation upon stimulation by comparable levels of agonist MCC peptides. In contrast, in our hands, T cells stimulated with CH27 cells prepulsed with either ER60-bio or β2m-bio showed very similar IL2 production to cells prepulsed with the control ER60scrbl-bio peptide (Figure 4B), a peptide which did not significantly alter the original endogenous peptide makeup. Therefore, on real APCs, agonist pMHCs appear to operate independently of endogenous pMHCs in T cell stimulation.

### TCR Triggering by Agonist pMHCs on Lipid Bilayers Requires T Cell Adhesion and Intact Cytoskeletal Function

**Figure 5:**
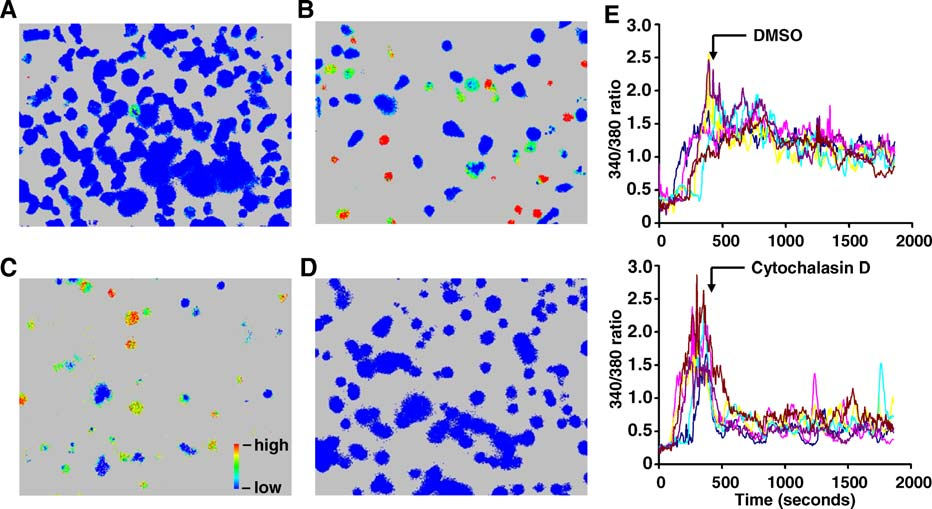
TCR Triggering by Monomeric Agonist pMHC Requires T Cell Adhesion to the Bilayer and Intact Actin Cytoskeletal Function (A and B) Lipid bilayers containing 5 mol% DOPE-biotin were anchored with monovalent (IEk-MCC)-SA. The bilayers were then used to stimulate T cells directly (A) or were further anchored with (ICAM-1)_2_-SA (B) before adding T cells. Bilayers anchored with only (ICAM-1-bio)_2_-SA did not induce T cell calcium flux. (C and D) A layer of CH27 cells pulsed for 1 h with 1 μM MCC-FITC was attached on cover glasses coated with poly-l-lysine. Fura-2 AM–pulsed AD10 T cells treated with 10 μM cytochalasin D for 1 h (D), or untreated AD10 T cells (C) were introduced, and calcium flux was monitored. (E) Similar to (C), except that 10 μM cytochalasin D (or DMSO as control) was introduced after T cell calcium flux reached peak levels.

Previous work [22] and our own experimental data argue strongly that monomeric agonist pMHC cannot trigger TCR in solution. To determine what confers the ability of a very small number of agonist pMHCs, anchored on a surface, to trigger TCR, we examined whether active adhesion was required for triggering by our artificial APCs. T cells spontaneously adhered to lipid bilayers containing NTA-Ni (unpublished data), probably due to surface charge interactions. The inability of this bilayer to become a nonadhesive surface made it unsuitable for studies of the role of adhesion in TCR triggering. Therefore, in order to have in hand a lipid bilayer system with controllable adhesion properties, we established a biotin-streptavidin–based artificial APC system (Figure S7). Streptavidin molecules specifically bind to POPC bilayers containing 5 mol% DOPE-biotin and diffuse freely (Figure S1C). IEk and ICAM-1 molecules were biotinylated at their AviTag sequences and mixed with streptavidin at ratios suitable for forming complexes with desired valencies. Monovalent (IEk-MCC)-streptavidin and divalent (ICAM-1)_2_-streptavidin complexes were further purified by gel filtration. T cell adhesion to this bilayer was strongly dependent on the presence of (ICAM-1)_2_-streptavidin on the bilayer (Videos S5 and S6). Bilayers anchored only with (IEk-MCC)-streptavidin failed to stimulate AD10 T cell calcium flux, even at relatively high ligand densities (1,400/mm^2^) (Figure 5A and Video S7). In contrast, in the presence of ICAM-1, bilayers anchored with (IEk-MCC)-streptavidin induced strong calcium flux (Figure 5B and Video S8), suggesting that adhesion is required for TCR triggering by agonist pMHC on the lipid bilayer.

In addition to adhesion, another important aspect of the dynamic 2D interface formed by two highly mobile cells, such as the T cell and its APC, is the impact of cytoskeletal movement, which may directly apply force upon pMHC-TCR binding. To test the role of the actin cytoskeleton in TCR triggering by agonist pMHC, AD10 T cells were treated with the actin depolymerizing agent, cytochalasin D, before interaction with a layer of CH27 cells pulsed with MCC peptides on glass. While untreated AD10 T cells responded with pronounced and sustained increases in intracellular calcium (Figure 5C and Video S9), AD10 T cells pretreated with cytochalasin D showed no response (Figure 5D and Video S10), although in both cases, there was contact between T cells and APCs (Figure S8). Similar results were observed when T cells were stimulated with IEk-MCC anchored on fluid lipid bilayers or plastic surfaces (Figure S9). Therefore, cytochalasin D treatment completely blunted T cell calcium responses to real or artificial APCs. To test whether actin cytoskeletal rearrangement is required for sustaining calcium flux that has already been initiated by real APCs, cytochalasin D was introduced after the calcium flux of untreated T cells reached peak levels. In agreement with a previous report [47], cytochalasin D quickly suppressed calcium flux (Figure 5E). Cytochalasin D treatment per se did not block events downstream of TCR triggering leading to T cell calcium flux, since calcium flux induced by anti-TCR antibody crosslinking was unaffected (Videos S11–S13). Cytochalasin D treatment also significantly reduced T cell mobility and morphological changes associated with interaction with CH27 cells (unpublished data). Intact actin cytoskeletal function is therefore required for TCR triggering by both artificial APCs and real APCs.

## Discussion

We demonstrate, using artificial APCs, that surface anchoring, adhesion, and actin cytoskeleton enable monomeric agonist pMHCs to trigger TCR with remarkable potency. T cells responded to an average of three and of 0.83 agonist pMHCs anchored on lipid bilayers and fixed plastic surfaces, respectively. Due to the nonaverage Poisson distribution of the ligands on the surfaces, the responding T cells might have contacted as many as seven and three agonist pMHCs, respectively (see Text S2). Although it is possible that T cells may have responded to a larger number of agonist pMHCs on lipid bilayers due to their diffusion and accumulation underneath T cells over time, the general agreement between the required numbers of ligands on lipid bilayers and plastic surfaces suggests that TCR can be triggered by a very low number of agonist pMHCs anchored on fluid or fixed surfaces.

**Figure 6:**
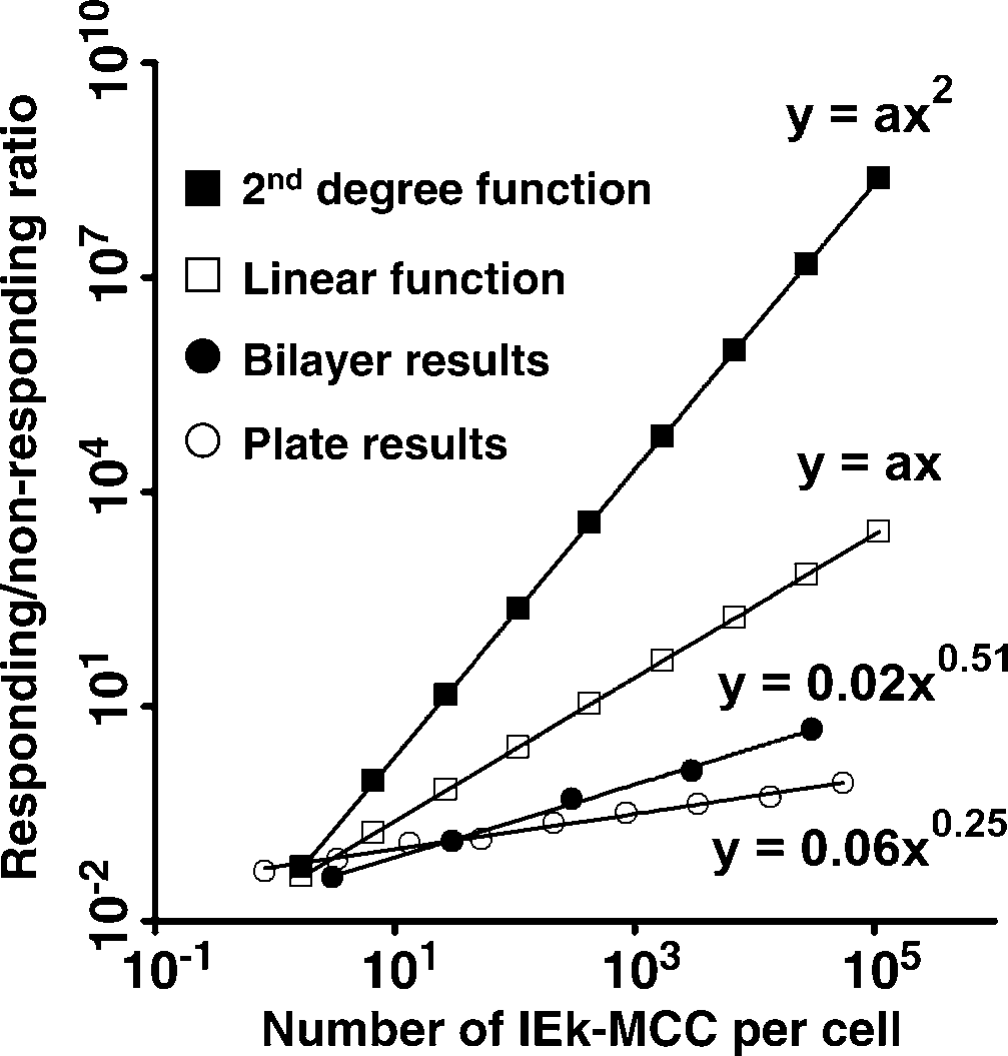
T Cells Respond to Agonist pMHC in a Sublinear Fashion AD10 T cell calcium responses to IEk-MCC diluted by IEk-HSP70 on lipid bilayers in Figure 2B and AD10 T cell IL2 responses to bio-IEk-MCC on streptavidin plates in Figure 3A are plotted as power functions of the calculated number of IEk-MCC per cell. T cell responses are shown as the ratio of responding to nonresponding cells. Also shown are theoretical curves of linear and second-degree function responses.

We believe that an alternative interpretation of our data, i.e., that TCR was triggered by agonist pMHC dimers, is unlikely for the following reasons. First, we took great caution in generating and purifying IEk-MCC proteins to minimize the existence of dimers in our protein preparation. The very low number of surface-anchored agonist pMHCs required for TCR triggering (<10 per cell) (Figures 2A–2C, 3A, and Text S2) makes it highly unlikely that TCR was triggered by dimer contamination in the protein preparation, which certainly did not contain >10% dimers (Figure S2A). Second, to exclude the possibility of “de facto” dimer formation by immobilization of two agonist pMHCs within close proximity by chance, we developed the lipid bilayer-based artificial APC system and went to great lengths to ensure the homogeneity and fluidity of the bilayer. Third, although dimers might, theoretically, form spontaneously between monomeric pMHCs diffusing freely on the bilayer, the necessary intrinsic affinity between MHC molecules is not supported by experimental evidence. Despite the availability of such highly sensitive techniques as surface plasmon resonance, no quantitative measurement of affinity between MHC molecules has been documented, suggesting that the affinity between MHC molecules is extremely low, if there is any. This is consistent with the fact that MHC-MHC dimers have not been observed in the vast majority of MHC crystallography studies [7,9–11,48–52]. The possibility of such a scenario is further reduced by the presence of a large number of “diluting” endogenous or null pMHCs. The lack of affinity between MHC molecules may not be relevant, if TCRs exist as dimers on the T cell and “recruit” two pMHC monomers sequentially. Whether TCRs are present as dimers on the T cell surface, however, is highly controversial [53–58]. Even if TCRs were dimers, the fast dissociation rate of agonist pMHC-TCR binding under force (discussed below) would make it very difficult to successfully recruit the second agonist pMHC molecule, especially when the number of agonist pMHCs is low. Finally, the shape of the T cell dose response curve further supports the sufficiency of monomeric pMHC for triggering. T cells responded to increasing numbers of IEk-MCC on the bilayer or on fixed surfaces in a sublinear fashion, as opposed to a second order dependence (*y* = *ax* ^2^) that would have indicated a requirement for IEk-MCC dimers (Figure 6). The sublinear response is consistent with the wide dynamic range observed in T cell responses to IEk-MCC on both lipid bilayers and plastic surfaces, and may indicate a progressive suppression of T cell sensitivity to increasing doses of agonist pMHCs. The physiological significance of this phenomenon remains to be investigated, but similar responses have been observed in other biological systems, such as the response of rod cells to photons triggering the photoreceptor, rhodopsin [59]. In summary, our data indicate that individual agonist pMHC monomers are sufficient to trigger TCR.

In a recent study [29], help from endogenous pMHCs was used to explain the remarkable T cell sensitivity to agonist pMHCs on real APCs. A pseudodimer model was proposed in which an endogenous pMHC engaging TCR could be bridged by CD4 to form a “pseudodimer” with an agonist pMHC engaging another TCR [29]. This model of TCR triggering was based upon experimental data showing that the very low TCR triggering capacity of agonist pMHC alone (requiring a density of more than 1,000 per cell on lipid bilayers) [29,60] was dramatically enhanced (more than 100-fold) by the presence of endogenous pMHCs. Our observation that one to ten agonist IEk-MCC alone triggered not only TCR-induced calcium flux, but also IL2 production, calls into question the purported requirement for help by endogenous pMHC. Indeed, using mouse T cells with a transgenic TCR of the same specificity, endogenous IEk-ER60 and null IEk-99A, which were shown to enhance TCR triggering by IEk-MCC in the previous report, did not, in our hands, enhance TCR triggering by IEk-MCC on fluid lipid bilayers or fixed plastic surfaces. As evidenced by our analyses using circular dichroism and peptide pulsing experiments, the lack of help by IEk-ER60 could not be attributed to incorrect protein folding or lack of peptide in the binding groove (Figure S2B–S2D). IEk-99A differs from IEk-MCC by only one residue, and the correctness of its tertiary structure is supported by the fact that tetrameric IEk-99A weakly binds T cells from AND TCR transgenic mice [35] and acts as a weak agonist for these T cells (Figure S10 and S. M. Hedrick, personal communication). The lack of help for TCR triggering by endogenous pMHC was further supported by our observation that biasing the endogenous peptide population on real APCs with ER60 or β2m peptides did not affect T cell activation by MCC peptides. This discrepancy may be partially explained by the different methodologies used in these studies. We consistently used MHC molecules with covalently linked peptides expressed in insect cells. In the previous report, both MHC with covalently linked peptides and empty MHC loaded with synthetic peptides were used, sometimes in the same experiment. The relative MHC occupancies by the different peptides were not determined. The differing results of T cell activation by real cells pulsed with peptides may be due to the fact that two totally different cell types were used in the two studies: previously, CHO cells expressing empty GPI-anchored IEk versus, in our study, CH27 B cells expressing IEk, as well as the appropriate adhesion and costimulatory molecules of real APCs. Again, in the previous study, the relative level of different peptides on the surface of CHO cells was not determined.

Theoretically, some aspects of the pseudodimer model of TCR remain to be reconciled with what has been learned regarding TCR triggering and pMHC-TCR interaction. The hypothesis that CD4 bridges the pseudodimer is not well supported by experimental data. Coreceptors bind MHC with very limited affinity and stability and with a fast off-rate [61]. In addition, CD4 has been shown to associate with TCR/CD3 via Lck and ZAP-70 in response to CD3 stimulation [62], but not on resting T cells. Another issue is that the role of endogenous pMHC is not clearly explained in the pseudodimer model. Intuitively, it seems that recruitment of free TCR-CD4 to an agonist pMHC-TCR binding pair would be easier than recruitment of the TCR-CD4 complex bound to endogenous pMHC anchored on an opposing membrane. The resulting complex should function equally well as a pseudodimer. Finally, using TAP-deficient cells, previous reports demonstrated that endogenous pMHCs had a negligible effect on the activation by agonist pMHCs of CD8^+^ T cells, which express the same TCR/CD3 complex [24,63].

What is it, then, that transforms a nonfunctional soluble monomeric agonist pMHC in solution into a powerful TCR triggering unit once it is attached to a surface? Recent data suggest that the mechanism of TCR triggering must include features unique to 2D binding. These include the need for two opposing membranes to reach the confinement length for association of membrane-anchored ligands and receptors, especially ones with small dimensions such as TCR and pMHC [31,32,64]. What has been omitted, however, is that a bound ligand–receptor pair, including pMHC-TCR, is constantly stressed and may be ruptured by forces from the active cytoskeletal rearrangement that supports the dynamic engagement between T cells and APCs. Recent in vivo studies using two-photon microscopy have characterized the interaction between T cells and antigen-loaded APCs in lymph nodes during the first 2 h as serial and dynamic, with T cells engaging and disengaging APCs at a speed of 5.4 μm/min [65,66]. T cells move with a velocity of 2.6 μm/min even at later stages of dynamic T cell/APC clustering. In this context, a single specific interaction between a TCR and an agonist pMHC may not be able to maintain the small, close membrane–membrane contact zone and keep tyrosine kinases and phosphatases segregated, as proposed in the kinetic-segregation model [30–32]. We demonstrate, in this study, that TCR triggering requires not only T cell adhesion to a surface, but also a functional actin cytoskeleton, consistent with the possibility that detaching forces are important in TCR triggering.

**Figure 7:**
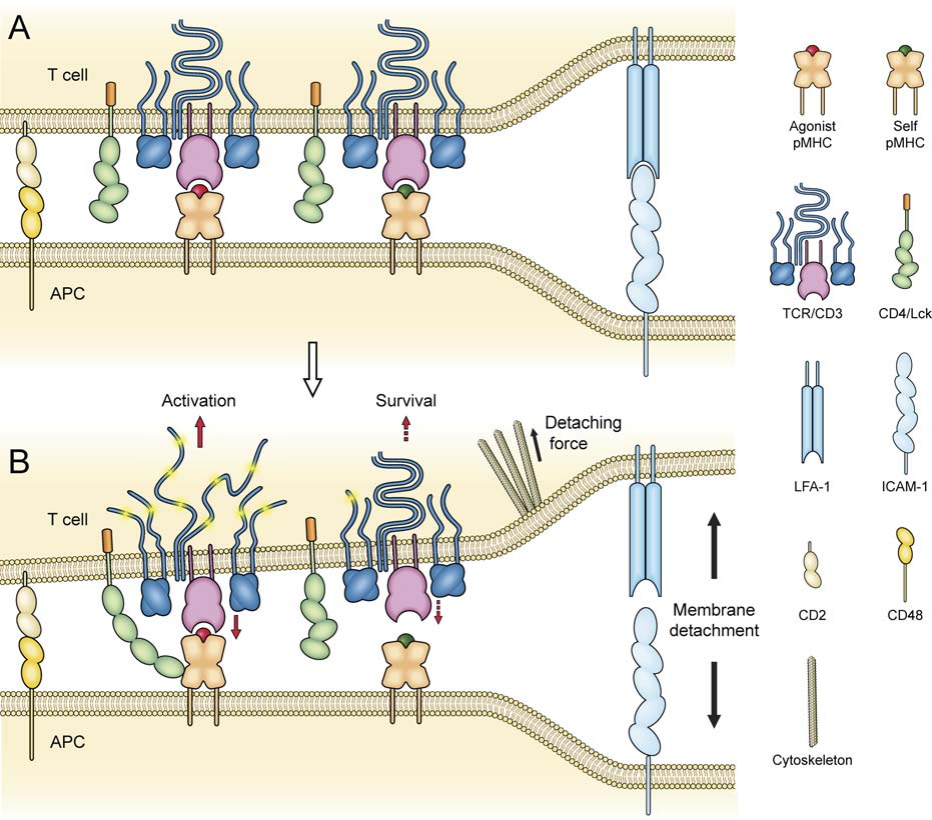
The Receptor Deformation Model of TCR Triggering (A) Interaction of pMHC and TCRs at the membrane–membrane contact site of the T cell/APC interface facilitated by adhesion molecules. The interaction between agonist pMHC and TCR per se does not initiate TCR signaling. (B) Forces generated from the cytoskeleton, which drive T cell morphological changes and active T cell movement relative to the APC, detach the T cell plasma membrane from the APC. Part of this force is delivered to the TCR/CD3 complex via pMHC-TCR binding. Binding between agonist pMHC and TCR is strong enough to deliver a force that deforms the TCR/CD3 complex to a conformation capable of initiating an activation signal. Weak binding between TCR and self pMHC breaks before such a force can be delivered. This binding could, however, deliver a weak force that causes minor receptor deformation, leading to a survival signal. The detaching force might also deform the MHC molecule to a conformation that binds coreceptors with higher affinity.

Here, taking into consideration the dynamic aspect of the 2D interaction between T cells and APCs, especially its likely impact on the dissociation of pMHC-TCR, we propose the receptor deformation model of TCR triggering (Figure 7). In this model, when a T cell encounters and scans an APC, pMHC and TCR interact when and where a sufficiently close membrane–membrane contact is formed. pMHC-TCR interaction per se, however, does not trigger TCR. A signal is initiated when the binding pair is pulled by a detaching force originating from cytoskeletal rearrangement, a force originally intended to detach the membrane–membrane contact for T cell movement. The pulling force induces a conformational change of the αβ TCR, which is then transferred to the intracellular domains of the CD3 complex through interactions between their extracellular domains and transmembrane domains [67]. Alternatively, it may directly cause changes in the relative positioning and orientation of the components of the TCR/CD3 complex. This pulling force may also induce conformational changes of the coreceptor binding domains of MHC molecules, and lead to better recruitment or conformational change of the coreceptors. The signal is initiated by increased access to and phosphorylation of ITAM domains of the CD3 complex by the coreceptor-associated tyrosine kinase, Lck. Therefore, the critical factor that determines whether a particular pMHC-TCR binding can lead to signaling is whether the binding has sufficient mechanical strength and appropriate kinetics to deliver an external force to TCR or pMHC to induce a conformational change, rather than the affinity, kinetics, or conformational change under zero force, as proposed in previous models.

The receptor deformation model provides a solution to the problem of how the binding between TCRs and pMHCs, both of which have hugely variable binding interfaces, leads to a uniform signal initiating conformational change of the TCR/CD3 complex. It also explains how T cells can detect an extremely low number of agonist pMHCs, alone, anchored on surfaces, as we observed in this study. Although under zero force, the half-life of binding (*t*_1/2_) of many agonist pMHC-TCR pairs has been reported to be more than 10 s, and as long as 50 s for strong agonists [68], it could be much shorter under a pulling force, because the dissociate rate (*k*_off_) increases exponentially with force [69]. Together, with the locomotion and dynamic morphological changes of the T cells, and the active lateral movement of TCR and pMHC on the fluid membranes [70], a fast dissociation could allow rapid triggering of multiple neighboring TCRs by a single agonist pMHC in a short period of time, leading to efficient temporal and spatial accumulation and integration of multiple signals, as previously proposed by the serial engagement model [71]. Furthermore, the receptor deformation model offers the “rupture force” of pMHC-TCR binding (the force needed to rupture the binding) as the mechanism by which T cells distinguish structurally similar agonist and endogenous pMHCs. This is supported by the excellent correlation found between rupture force and the zero force *k*_off_ [72], which is correlated with TCR triggering by pMHC [68]. It is intriguing to speculate that weak rupture forces between TCR and endogenous pMHC may support minor changes of the TCR/CD3 complex and generate survival signals. Finally, this model is consistent with experimental evidence that cognate pMHC-TCR interaction induces a conformational change in the CD3 complex [73,74]. Also, force-induced conformational change has been documented in other ligand-receptor systems, such as the conformational change of LFA-1 α domain [75] and Escherichia coli adhesin FimH [76] in response to shear forces.

The receptor deformation model is very different from a previously proposed raft-TCR collision model that also involves force, in which the movement of lipid rafts on T cells driven by actin or shear stress promotes collision of raft-associated Lck and the TCR/CD3 complex [77]. This model works through increased kinase-TCR proximity, rather than conformational change. In the raft-TCR collision model, the force is applied laterally on the lipid raft and does not impact the binding kinetics of pMHC-TCR interaction. Our model is also distinct from the permissive geometry model, which proposes that TCRs are present as oligomeric clusters on T cells, and that binding of multimeric agonist pMHCs with a certain permissive geometry triggers TCRs through changes in the relative positioning of individual TCRs within the cluster [57,78]. The key differences are that changes in TCR geometry are induced solely by pMHC-TCR interaction, without the requirement of external mechanical force, and that multimeric pMHCs are required for triggering.

Notwithstanding its uniqueness and advantages, the receptor deformation model needs to be reconciled with the well-documented observation that soluble multimeric agonist pMHCs trigger TCR [22,79]. In our opinion, the fact that T cells can be activated via a receptor crosslinking mechanism used by B cells is not surprising, given their similarity in evolution, development, antigen receptor structure, and intracellular signaling pathways. Our work does not exclude the possibility that when agonist pMHCs are present at extremely high levels on APCs, TCR crosslinking is a functional mechanism of TCR triggering. In this situation, the chance of two neighboring MHC molecules, both presenting agonist peptides, may be reasonably high. This, and the fact that a high proportion of MHC molecules on the APC surface is immobile [26,27], make it plausible that two monomeric agonist pMHCs might be stably localized in close enough proximity to act as functional dimers capable of crosslinking TCRs. Given the rarity of agonist pMHCs on APCs under physiological conditions, however, a mechanism that does not rely on TCR crosslinking, such as receptor deformation, is likely to be the main working mechanism of TCR triggering.

In conclusion, we provide strong evidence that extremely small numbers (<10 per cell) of surface-anchored agonist pMHCs trigger TCR, independent of endogenous pMHCs, but dependent upon adhesion and intact cytoskeletal function. We propose the receptor deformation model, in which cytoskeletal rearrangement delivers the driving force for TCR triggering. Given its merits in providing a straightforward mechanism of TCR triggering and in explaining both the high sensitivity and the high specificity of agonist pMHC detection by T cells, we believe that this model warrants further investigation.

## Materials and Methods

### Mice, chemicals, constructs, and antibodies.

B10.BR H-2^k^ mice and 5C.C7 mice were purchased from Jackson Laboratories and Taconic, respectively. AD10 and AND TCR transgenic mice were provided by J. W. Kappler (National Jewish Medical and Research Center). T cell blasts were generated by a mixed culture of splenocytes from TCR transgenic mice and B10.BR mice in Click's medium supplemented with 10% fetal calf serum (FCS), 2 mM l-glutamine, 50 μM β-mercaptoethanol, penicillin/streptomycin, 1 mM sodium pyruvate, 0.1 mM nonessential amino acids, and 50 μg/ml pigeon cytochrome c. T cell blasts were used on day 7 to day 9 poststimulation. POPC, DOPE-biotin, DOPE-NBD, and DOGS-NTA were purchased from Avanti Polar Lipids; 14-4-4s mAb was generated from a hybridoma kindly provided by J. W. Kappler. Anti-ICAM-1 monoclonal antibody, YN1.7.4, was produced from a hybridoma obtained from ATCC. Anti-leucine zipper antibody, 2H11, was produced from a hybridoma from E. L. Reinherz (Dana-Farber Cancer Institute). Streptavidin was from Sigma. APC-labeled anti-mouse IL2 antibody, JES6-5H4, and APC-labeled anti-mouse IL4 antibody, 11H11, were from BD Biosciences. The D10.IL2 T cell line, CH27, and M12.C3 B cell lymphoma cells were provided by A. Kupfer (Johns Hopkins University), J. Monroe (University of Pennsylvania), and S. Ostrand-Rosenberg (University of Maryland), respectively. ER60-bio, β2m-bio, ER60scrbl-bio (APPNIKYFLSFGTK), and MCC-FITC peptides were synthesized by Genemed Synthesis. Baculovirus transfer vector, pBlueBac4.5/V5-His, Sf9, and Hi5 insect cells were purchased from Invitrogen.

### Protein expression and purification.

All proteins were expressed in secreted form by infecting Hi5 insect cells with baculovirus. Baculovirus transfer vector for ICAM-1-AviTag-HisTag was constructed with pBlueBac4.5/V5-His and murine ICAM-1 cDNA amplified from mouse spleen mRNA. Transfer vectors for IEk-MCC, IEk-HSP70, IEk-ER60, IEk-99A, and IAk-CA with AviTag and HisTag were constructed based on a transfer vector for IEk-MCC-AviTag (a gift from J. W. Kappler). cDNA encoding IAk-CA stabilized with a leucine zipper was provided by E. L. Reinherz. ICAM-1, IEk, and IAk proteins were affinity purified with columns conjugated with NTA-Ni, 14-4-4s antibody, and 2H11 antibody, respectively. Each protein was then further purified by gel filtration with a Superdex 200 10/300 GL column (Amersham Biosciences) before storage. IEk-MCC was kept at 4 °C for less than 10 d and was never frozen and thawed. An additional round of gel filtration was always performed immediately before use of the proteins in experiments. IEk-MCC and ICAM-1 were biotinylated with BIRA enzyme (Avidity) and purified by gel filtration. The biotinylation rate was about 50%, as measured by an ELISA-based assay. To generate (IEk-MCC)-SA, bio-IEk-MCC and streptavidin were mixed at a 1:5 molar ratio, and monovalent complexes were purified by two successive rounds of gel filtration with a Superdex 200 10/300 GL column. To generate (ICAM-1)_2_-SA, bio-ICAM-1 and streptavidin were mixed at a 1:1 molar ratio, and bivalent complexes were purified by gel filtration. Purified complexes were used immediately without storage.

### Lipid bilayer preparation.

Liposomes were prepared by sonication using POPC mixed with 0.2 mol% DOPE-NBD and 5 mol% DOGS-NTA or DOPE-biotin in HEPES-Na buffer (150 mM NaCl, 5 mM HEPES [pH 7.4] with 0.02% sodium azide). The liposome solution was then spun at 40,000 rpm for 2 h, and small unilamellar vesicles (SUV) were isolated by taking the top four-fifths of the solution. Supported planer lipid bilayers were prepared by liposome fusion for 10 min at 4 °C on glass cover slips that had been extensively cleaned with Contrad70 detergent (Decon Laboratories) and chromic sulfuric acid solution (Sigma). Half of the lipid bilayer was collapsed by exposure to air before transferring to 25 °C for 40 min for expansion. Lipid bilayers were then transferred under HEPES-Na buffer into FCS2 flow chambers (Bioptechs) for imaging. For bilayer containing DOGS-NTA, HEPES-Na buffer containing 0.25 mM NiSO_4_ was introduced into the chamber for 10 min to charge DOGS-NTA with Ni^2+^.

### Lipid bilayer-based artificial APC preparation.

Lipid bilayers were blocked with blocking buffer (5 mg/ml BSA in HEPES-Na buffer [pH 7.4]) for 10 min. For lipid bilayers with DOGS-NTA, 10 μg/ml IEk and 1 μg/ml ICAM-1 in blocking buffer were introduced into the chamber and incubated for 30 min at 25 °C. The bilayer was then washed with imaging buffer (150 mM NaCl, 5 mM KCl, 2 mg/ml glucose, 10 mM HEPES [pH 7.4], 33 mg/ml BSA) containing 1 mM CaCl_2_ and 1 mM MgCl_2_ for 3 min under constant buffer flow. For bilayers with DOPE-biotin, the blocked bilayer was incubated with 1 μg/ml (IEk-MCC)-SA first, followed by 5 μg/ml (ICAM-1)_2_-SA in imaging buffer. The bilayer was then washed with imaging buffer containing 0.25 mM MnCl_2_ and 0.25 mM CaCl_2_.

### Determination of IEk ligand density on lipid bilayers.

For POPC bilayers with 5 mol% DOGS-NTA, IEk-MCC was labeled with FITC. For POPC bilayers with 5 mol% DOPE-biotin, biotinylated IEk-MCC was labeled with FITC and was used to form the (FITC-IEk-MCC)-SA complex. After binding of the FITC-labeled proteins, the bilayers were lysed with 200 μl of DPBS (pH 7.4) containing 1% Triton-X100 and 0.5 mg/ml BSA. The intensity of FITC was read using a high numerical aperture (0.5) 10× air objective (Zeiss) and recorded with a 12-bit cooled CCD camera. POPC bilayer without DOGS-NTA or DOPE-bio was used as a blank control. After subtracting the FITC intensity of the blank, the FITC intensity was translated into protein concentration based on a standard curve generated using FITC-labeled proteins of known concentration. The ligand densities were calculated based on the amount of protein bound and the area of the lipid bilayer.

### Microscopy and image analysis

For calcium imaging, 5 × 10^6^ TCR transgenic CD4^+^ T cells were pulsed with 5 μM fura-2-AM (Invitrogen) for 30 min at room temperature and washed twice with imaging buffer before introduction into the flow chamber. The 510-nm emissions excited by 380 nm and 340 nm were captured at 5-s intervals for 30 min using a 40× oil Plan-Neofluar objective on an Axioplan2 Microscope (Zeiss) at 25 °C. Data were collected and analyzed with SlideBook software (Intelligent Imaging Innovations). Nomarski DIC bright-field imaging with a 60× water Achroplan objective was used to capture cell adhesion on the lipid bilayer and to measure the T cell diameter. For FRAP, a pattern of 4-μm radius was photobleached with a 3 mW nitrogen laser (Stanford Research Systems), and the recovery was captured using an attenuated xenon light source (Sutter).

### T cell stimulation on plates and intracellular IL2 staining.

Bio-IEk-MCC in PBS pH 8.0 was coated on Immulon2 U-bottom ELISA plates (Thermo Electron) or SigmaScreen streptavidin-coated plates (Sigma; binding capacity ≥6 pmol biotin/well) for 18 h at 4 °C or 37 °C, respectively. For experiments requiring saturation of the plate by another coating protein, 10 μg/ml nonbiotinylated or biotinylated proteins were subsequently added to ELISA or streptavidin plates in a 50 or 75 μl volume, respectively. The ELISA or streptavidin plate was then incubated at room temperature for 4 h or 1 h, respectively. Plates were washed, and 2.5 × 10^5^ T cells were added in complete Click's medium containing 20 μg/ml brefeldin A. After 7 h incubation, cells were harvested, fixed with 3% formaldehyde in PBS, permeabilized with PBS buffer containing 1% BSA and 0.1% saponin, and stained with anti-IL2 mAb for flow cytometric analysis.

### Pulsing of CH27 cells with peptides.

CH27 cells at 5 × 10^5^/ml were incubated with 1 mM β2m-bio, ER60-bio, or Er60srcbl-bio peptides in Click's medium with 10 mM HEPES for 20 h at 37 °C. After washing three times with Click's medium, cells were incubated for 30 min at 37 °C with MCC-FITC peptides at different dilutions. After washing, half of the cells pulsed at each dilution were then stained with streptavidin-Cy5, and the FITC and Cy5 intensities were measured by flow cytometry. The other half of the cells were incubated with AD10 T cells at a 1:1 ratio for assay of IL2 production.

## Supporting Information

Figure S1 Long-Range Free Diffusion of Glass-Supported Lipid Bilayers and Anchored Proteins (A) Fluorescence recovery of a photobleached area on the POPC/1mol% DOPE-NBD bilayer.
(B) Recovery of a photobleached area on the POPC bilayer containing 5mol% DOGS-NTA-Ni with bound FITC-labeled IEk-MCC.
(C) Recovery of a photobleached area on the POPC bilayer containing 5mol% DOPE-biotin with bound FITC-labeled streptavidin. The bilayer itself was not fluorescently labeled in (B) and (C). The scale bar represents 10 μm.
(1.2 MB DOC)

Figure S2 Characterization of the Purified IEk Proteins with Covalently Linked Peptides Expressed and Secreted by Insect Cells (A) FPLC gel filtration chromatography of IEk-MCC and BSA. A single peak corresponding to IEk-MCC monomer was observed. Only fractions from the center of the peak were collected for anchoring onto the lipid bilayer. As a comparison, the chromatography of similar-sized BSA shows naturally occurring dimers and multimers. The percentages of BSA monomers, dimmers, and multimers were calculated based on the areas of the peaks.
(B) Circular dichroism spectra of purified IEk-MCC, IEk-ER60, and IEk-HSP70 proteins (300 μg/ml) at 25 °C. Data were acquired using a 0.1-cm cuvette on a J-810 CD spectropolarimeter (JASCO) with 0.2-nm steps and 2-s integration times. Each spectrum is the average of three scans.
(C) Thermal melt of IEk proteins monitored at 208 nm. Temperature was increased at 2 °C intervals with 100-s equilibration times for each temperature point. Each melting curve is the average of three melts. The higher melting temperature than previously reported for IEk with bound peptide and the “kink” in the middle of melting curve may be attributed to the fact that all peptides in this study are covalently linked to IEk. The covalent linkage may enhance stabilization of the α-helical structure, which forms the IEk peptide-binding groove, by bound peptides.
(D) Bio-IEk-ER60 and bio-IEk-HSP70 cannot be loaded with MCC peptides. Bio-IEk-ER60 and bio-IEk-HSP70 proteins were incubated with 50 μM MCC peptides in PBS-citric acid buffer (pH 5.3) at 37 °C for 18 h on streptavidin-coated plates. The plates were then washed and AD10 T cells were added in medium containing 20 μg/ml brefeldin A. IL2 production was measured by intracellular staining after 7 h.
(76 KB DOC)

Figure S3 IAk-CA Activates D10 T Cells ELISA plates coated with 30 μg/ml IAk-CA or BSA were used to stimulate D10.IL2 T cells in medium containing 20 μg/ml brefeldin A for 7 h. IL4 production was measured by intracellular staining with the monoclonal antibody, 11H11, by flow cytometry.
(97 KB DOC)

Figure S4 The Level of bio-IEk-MCC Proteins on the ELISA Plates Used in Figure 3D Was Determined by Binding of SA-HRP The amount of bound SA-HRP was determined by measuring the optical density at 415 nm (OD415 nm) from ABTS substrate colorization.
(50 KB DOC)

Figure S5 Determining the Streptavidin Binding Capacity of 14-4-4s-bio Antibody Using a Gel Filtration Chromatography-Based Assay Ten micrograms of 14-4-4s-bio (Ab) was mixed with excess amounts of streptavidin (SA). The gel filtration plot (using the same Superdex 200 10/300 GL column) of the mixture (Ab-SA) was superimposed by the plots of known amounts of SA and Ab. The amount of SA and the SA:Ab ratio in the Ab-SA complex was calculated to be 4.67:1, based on the integrated areas of Ab-SA, Ab, and SA. These data are representative of two independent experiments.
(48 KB DOC)

Figure S6 Measuring the Level of Endogenous Peptides on CH27 and M12.C3 Cells after Peptide Pulsing CH27 or M12.C3 cells incubated with 1 mM β2m-bio, ER60-bio, or ER60scrbl-bio for 20 h were washed and stained with streptavidin-Cy5. To measure the level of IEk, CH27 cells were stained with IEk-specific antibody 14-4-4s-bio followed by streptavidin-Cy5. The fluorescence intensity of Cy5 was measured by flow cytometry. The peptide occupancy of IEk was calculated based on Cy5 intensities on biotinylated peptide-pulsed cells relative to those on cells stained with antibody 14-4-4s-bio, and the streptavidin binding capacity of the antibody (as calculated in Figure S5).
(192 KB DOC)

Figure S7 Schematics of the Interaction between the T Cell and the Biotin Lipid Bilayer-Based Artificial APC Monovalent (IEk-MCC)-SA and bivalent (ICAM-1)_2_-SA were generated and purified by gel filtration. Lipid bilayers containing 5 mol% DOPE-biotin were anchored with (IEk-MCC)-SA alone (A) or together with (ICAM-1)_2_-SA (B).
(87 KB DOC)

Figure S8 Contact of MCC-Pulsed CH27 Cells and AD10 T Cells with or without Treatment with Cytochalasin D The green cells are T cells. Images were taken after calcium flux monitoring, as described in Figure 5C and 5D.
(452 KB DOC)

Figure S9 TCR Triggering by Agonist pMHC on Lipid Bilayers and Plastic Surfaces Requires Intact Actin Cytoskeletal Function (A and B) Lipid bilayers containing 5 mol% DOGS-NTA-Ni were incubated with 1 μg/ml IEk-MCC. After washing, the bilayers were used to stimulate fura-2–pulsed AD10 T cells treated with 10 μM cytochalasin D for 1 h (B), or untreated T cells (A). Calcium flux was monitored using a 40× oil objective and displayed in pseudocolor.
(C and D) The 12-well tissue culture plates were coated with 100 μg/ml streptavidin in PBS (pH 7.4) for 2 h at 37 °C. After washing, the plates were blocked with PBS (pH 7.4) containing 5 mg/ml BSA for 10 min and incubated with 1 μg/ml bio-IEk-MCC for 4 h at room temperature. After washing, the plate was used to stimulate fura-2–pulsed AD10 T cells treated with 10 μM cytochalasin D for 1 h (D), or untreated T cells (C). Calcium flux was monitored using a 60× water immersion objective and displayed in pseudocolor.
(287 KB DOC)

Figure S10 IEk-99A Is a Weak Agonist for AND TCR Transgenic T Cells ELISA plates coated with 10 μg/ml IEk-MCC or IEk-99A were used to stimulate primed AND T cells in medium containing 20 μg/ml brefeldin A for 7 h. IL2 production was measured by intracellular staining and flow cytometry.
(27 KB DOC)

Text S1 Possible Reasons for the Difference in Shape and Range of the Response Curves to IEk on Streptavidin versus ELISA Plates (26 KB DOC)

Text S2 Calculation Based on Poisson Distribution of the Number of pMHCs That Activated 5% of T Cells on Plastic Plates or Lipid Bilayers (38 KB DOC)

Video S1 Time-Lapse Video Corresponding to Image in Figure 2A: IEk-MCC Mixed with GFP-HisTag NTA-Ni bilayers were anchored with IEk-MCC mixed with GFP-HisTag at a 1:1,000 ratio. Calcium responses of fura-2–pulsed AD10 CD4^+^ T cells were captured at 5-s intervals for 30 min. The level of intracellular calcium is displayed in pseudocolor. The speed of the video is 25 times real time.
(8.5 MB MOV)

Video S2 Time-Lapse Video Corresponding to Image in Figure 2A: Control Only GFP-HisTag was anchored on the NTA-Ni bilayer. Calcium responses of fura-2–pulsed AD10 CD4^+^ T cells were captured at 5-s intervals for 30 min. The level of intracellular calcium is displayed in pseudocolor. The speed of the video is 25 times real time.
(6 MB MOV)

Video S3 Time-Lapse Video Corresponding to Image in Figure 2A: IEk-MCC Mixed with IEk-ER60 NTA-Ni bilayers were anchored with IEk-MCC mixed with IEk-ER60 at a 1:1,000 ratio. Calcium responses of fura-2–pulsed AD10 CD4^+^ T cells were captured at 5-s intervals for 30 min. The level of intracellular calcium is displayed in pseudocolor. The speed of the video is 25 times real time.
(4.6 MB MOV)

Video S4 Time-Lapse Video Corresponding to Image in Figure 2A: IEk-MCC Mixed with IEk-HSP70 NTA-Ni bilayers were anchored with IEk-MCC mixed with IEk-HSP70 at a 1:1,000 ratio. Calcium responses of fura-2–pulsed AD10 CD4^+^ T cells were captured at 5-s intervals for 30 min. The level of intracellular calcium is displayed in pseudocolor. The speed of the video is 25 times real time.
(3.6 MB MOV)

Video S5 T cells Adhere to Bilayers with Anchored ICAM-1 POPC bilayers with 5 mol% DOPE-biotin, with anchored (ICAM-1)_2_-SA, were tilted at a very small angle so that, without adhesion (Video S6), T cells moved slowly on the surface due to gravity. T cells were imaged in buffer containing 0.25 mM Ca^2+^ and 0.25 mM Mn^2+^ to activate LFA-1 and promote adhesion. Movement of T cells was captured with DIC bright field imaging at 5-s intervals. The speed of the video is 50 times real time.
(1.3 MB MOV)

Video S6 T cells Do Not Adhere to Bilayers without Anchored ICAM-1 POPC bilayers with 5 mol% DOPE-biotin, without anchored (ICAM-1)_2_-SA, were tilted at a very small angle so that, without adhesion, T cells moved slowly on the surface due to gravity. T cells were imaged in buffer containing 0.25 mM Ca^2+^ and 0.25 mM Mn^2+^ to activate LFA-1 and promote adhesion. Movement of T cells was captured with DIC bright field imaging at 5-s intervals. The speed of the video is 50 times real time.
(2.7 MB MOV)

Video S7 Time-Lapse Video Corresponding to Figure 5A Calcium responses of fura-2–pulsed AD10 CD4^+^ T cells were captured at 5-s intervals for 10 min. Calcium levels are displayed in pseudocolor. The speed of the video is 50 times real time. Due to the quenching effect of Mn^2+^ in the imaging buffer on the fluorescence of fura-2, fura-2–pulsed AD10 T cells gradually lost fluorescence intensity during the course of image capture. Note that the few T cells with continuously high calcium levels are dead cells.
(1.2 MB MOV)

Video S8 Time-Lapse Video Corresponding to Figure 5B Calcium responses of fura-2–pulsed AD10 CD4^+^ T cells were captured at 5-s intervals for 10 min. Calcium levels are displayed in pseudocolor. The speed of the video is 50 times real time. Due to the quenching effect of Mn^2+^ in the imaging buffer on the fluorescence of fura-2, fura-2–pulsed AD10 T cells gradually lost fluorescence intensity during the course of image capture. Note that the few T cells with continuously high calcium levels are dead cells.
(1 MB MOV)

Video S9 Cytochalasin D Treatment of T Cells Blocks Calcium Flux Induced by CH27 Cells Pulsed with MCC: Results for Untreated Cells A layer of CH27 cells pulsed for 1 h with 1 μM MCC-FITC was attached on cover glasses coated with poly-l-lysine. Fura-2 AM–pulsed untreated AD10 T cells were introduced, and calcium flux was monitored. Calcium levels are displayed in pseudocolor. The speed of the video is 50 times real time.
(3.9 MB MOV)

Video S10 Cytochalasin D Treatment of T Cells Blocks Calcium Flux Induced by CH27 Cells Pulsed with MCC A layer of CH27 cells pulsed for 1 h with 1 μM MCC-FITC were attached on cover glasses coated with poly-l-lysine. AD10 T cells treated for 1 h with 10 μM cytochalasin D were introduced, and calcium flux was monitored. Calcium levels are displayed in pseudocolor. The speed of the video is 50 times real time.
(5 MB MOV)

Video S11 Cytochalasin D Treatment Does Not Affect Calcium Flux Stimulated by Antibody Crosslinking: Results of Untreated Cells Untreated AD10 T cells were attached on poly-l-lysine–coated cover glasses in a flow chamber; 10 μg/ml biotinylated anti-V_β_3 monoclonal antibody, KJ25, was introduced and incubated for 10 min. A few T cells fluxed calcium at low levels during incubation. The antibody was then washed away, and 10 μg/ml streptavidin was introduced. Crosslinking by streptavidin led to high-level calcium flux in most of the cells (the last quarter of the video). Calcium levels are displayed in pseudocolor. The speed of the video is 50 times real time.
(5.7 MB MOV)

Video S12 Cytochalasin D Treatment Does Not Affect Calcium Flux Stimulated by Antibody Crosslinking AD10 T cells treated with 10 μM cytochalasin D for 1 h at 37 °C were attached on poly-l-lysine–coated cover glasses in a flow chamber; 10 μg/ml biotinylated anti-V_β_3 monoclonal antibody, KJ25, was introduced and incubated for 10 min. A few T cells fluxed calcium at low levels during incubation. The antibody was then washed away, and 10 μg/ml streptavidin was introduced. Crosslinking by streptavidin led to high-level calcium flux in most of the cells (the last quarter of the video). Calcium levels are displayed in pseudocolor. The speed of the video is 50 times real time.
(5.1 MB MOV)

Video S13 Cytochalasin D Treatment Does Not Affect Calcium Flux Stimulated by Antibody Crosslinking: Results of Cytochalasin D Introduced Together with Streptavidin Untreated AD10 T cells were attached on poly-l-lysine–coated cover glasses in a flow chamber. The untreated AD10 T cells were incubated with KJ25 antibody first, then 10 μg/ml streptavidin was introduced together with 10 μM cytochalasin D. A few T cells fluxed calcium at low levels during incubation. Crosslinking by streptavidin led to high-level calcium flux in most of the cells (the last quarter of the video). Calcium levels are displayed in pseudocolor. The speed of the video is 50 times real time.
(6.7 MB MOV)

## Abbreviations

APC: antigen-presenting cell
IL2: interleukin 2
MHC: major histocompatibility complex
NTA: nitrilo triacetic acid
pMHC: peptide–major histocompatibility complex
TCR: T cell receptor
