# Supplementary material for: Surface-Anchored Monomeric Agonist pMHCs Alone Trigger TCR with High Sensitivity
Source: PLoS Biol. 2008 Feb 26;6(2):e43. doi: 10.1371/journal.pbio.0060043 (PMC2253636; doi:10.1371/journal.pbio.0060043)
Supplement: Figure S1 — (A) Fluorescence recovery of a photobleached area on the POPC/1mol% DOPE-NBD bilayer. (B) Recovery of a photobleached area on the POPC bilayer containing 5mol% DOGS-NTA-Ni with bound FITC-labeled IEk-MCC. (C) Recovery of a photobleached area on the POPC bilayer containing 5mol% DOPE-biotin with bound FITC-labeled streptavidin. The bilayer itself was not fluorescently labeled in (B) and (C). The scale bar represents 10 μm. (1.2 MB DOC) [file pbio.0060043.sg001.doc]

**Figure S1 (2 column-widths)**
