# Supplementary material for: Surface-Anchored Monomeric Agonist pMHCs Alone Trigger TCR with High Sensitivity
Source: PLoS Biol. 2008 Feb 26;6(2):e43. doi: 10.1371/journal.pbio.0060043 (PMC2253636; doi:10.1371/journal.pbio.0060043)
Supplement: Figure S2 — (A) FPLC gel filtration chromatography of IEk-MCC and BSA. A single peak corresponding to IEk-MCC monomer was observed. Only fractions from the center of the peak were collected for anchoring onto the lipid bilayer. As a comparison, the chromatography of similar-sized BSA shows naturally occurring dimers and multimers. The percentages of BSA monomers, dimmers, and multimers were calculated based on the areas of the peaks. (B) Circular dichroism spectra of purified IEk-MCC, IEk-ER60, and IEk-HSP70 proteins (300 μg/ml) at 25 °C. Data were acquired using a 0.1-cm cuvette on a J-810 CD spectropolarimeter (JASCO) with 0.2-nm steps and 2-s integration times. Each spectrum is the average of three scans. (C) Thermal melt of IEk proteins monitored at 208 nm. Temperature was increased at 2 °C intervals with 100-s equilibration times for each temperature point. Each melting curve is the average of three melts. The higher melting temperature than previously reported for IEk with bound peptide and the “kink” in the middle of melting curve may be attributed to the fact that all peptides in this study are covalently linked to IEk. The covalent linkage may enhance stabilization of the α-helical structure, which forms the IEk peptide-binding groove, by bound peptides. (D) Bio-IEk-ER60 and bio-IEk-HSP70 cannot be loaded with MCC peptides. Bio-IEk-ER60 and bio-IEk-HSP70 proteins were incubated with 50 μM MCC peptides in PBS-citric acid buffer (pH 5.3) at 37 °C for 18 h on streptavidin-coated plates. The plates were then washed and AD10 T cells were added in medium containing 20 μg/ml brefeldin A. IL2 production was measured by intracellular staining after 7 h. (76 KB DOC) [file pbio.0060043.sg002.doc]

**Figure S2 (~1.7 column-widths)**
