# Supplementary material for: Surface-Anchored Monomeric Agonist pMHCs Alone Trigger TCR with High Sensitivity
Source: PLoS Biol. 2008 Feb 26;6(2):e43. doi: 10.1371/journal.pbio.0060043 (PMC2253636; doi:10.1371/journal.pbio.0060043)
Supplement: Figure S3 — ELISA plates coated with 30 μg/ml IAk-CA or BSA were used to stimulate D10.IL2 T cells in medium containing 20 μg/ml brefeldin A for 7 h. IL4 production was measured by intracellular staining with the monoclonal antibody, 11H11, by flow cytometry. (97 KB DOC) [file pbio.0060043.sg003.doc]

**Figure S3 (1 column-width)**
