# Supplementary material for: Surface-Anchored Monomeric Agonist pMHCs Alone Trigger TCR with High Sensitivity
Source: PLoS Biol. 2008 Feb 26;6(2):e43. doi: 10.1371/journal.pbio.0060043 (PMC2253636; doi:10.1371/journal.pbio.0060043)
Supplement: Figure S4 — The amount of bound SA-HRP was determined by measuring the optical density at 415 nm (OD415 nm) from ABTS substrate colorization. (50 KB DOC) [file pbio.0060043.sg004.doc]

**Figure S4 (1 column-width)**
