# Supplementary material for: Surface-Anchored Monomeric Agonist pMHCs Alone Trigger TCR with High Sensitivity
Source: PLoS Biol. 2008 Feb 26;6(2):e43. doi: 10.1371/journal.pbio.0060043 (PMC2253636; doi:10.1371/journal.pbio.0060043)
Supplement: Figure S5 — Ten micrograms of 14-4-4s-bio (Ab) was mixed with excess amounts of streptavidin (SA). The gel filtration plot (using the same Superdex 200 10/300 GL column) of the mixture (Ab-SA) was superimposed by the plots of known amounts of SA and Ab. The amount of SA and the SA:Ab ratio in the Ab-SA complex was calculated to be 4.67:1, based on the integrated areas of Ab-SA, Ab, and SA. These data are representative of two independent experiments. (48 KB DOC) [file pbio.0060043.sg005.doc]

**Figure S5 (2 column-widths)**
