# Supplementary material for: Surface-Anchored Monomeric Agonist pMHCs Alone Trigger TCR with High Sensitivity
Source: PLoS Biol. 2008 Feb 26;6(2):e43. doi: 10.1371/journal.pbio.0060043 (PMC2253636; doi:10.1371/journal.pbio.0060043)
Supplement: Figure S6 — CH27 or M12.C3 cells incubated with 1 mM β2m-bio, ER60-bio, or ER60scrbl-bio for 20 h were washed and stained with streptavidin-Cy5. To measure the level of IEk, CH27 cells were stained with IEk-specific antibody 14-4-4s-bio followed by streptavidin-Cy5. The fluorescence intensity of Cy5 was measured by flow cytometry. The peptide occupancy of IEk was calculated based on Cy5 intensities on biotinylated peptide-pulsed cells relative to those on cells stained with antibody 14-4-4s-bio, and the streptavidin binding capacity of the antibody (as calculated in Figure S5). (192 KB DOC) [file pbio.0060043.sg006.doc]

**Figure S6 (2 column-widths)**
