# Supplementary material for: Surface-Anchored Monomeric Agonist pMHCs Alone Trigger TCR with High Sensitivity
Source: PLoS Biol. 2008 Feb 26;6(2):e43. doi: 10.1371/journal.pbio.0060043 (PMC2253636; doi:10.1371/journal.pbio.0060043)
Supplement: Figure S7 — Monovalent (IEk-MCC)-SA and bivalent (ICAM-1)2-SA were generated and purified by gel filtration. Lipid bilayers containing 5 mol% DOPE-biotin were anchored with (IEk-MCC)-SA alone (A) or together with (ICAM-1)2-SA (B). (87 KB DOC) [file pbio.0060043.sg007.doc]

**Figure S7 (2 column-widths)**
