# Supplementary material for: Surface-Anchored Monomeric Agonist pMHCs Alone Trigger TCR with High Sensitivity
Source: PLoS Biol. 2008 Feb 26;6(2):e43. doi: 10.1371/journal.pbio.0060043 (PMC2253636; doi:10.1371/journal.pbio.0060043)
Supplement: Figure S8 — The green cells are T cells. Images were taken after calcium flux monitoring, as described in Figure 5C and 5D. (452 KB DOC) [file pbio.0060043.sg008.doc]

**Figure S8 (1.5 column-widths)**
