# Supplementary material for: Surface-Anchored Monomeric Agonist pMHCs Alone Trigger TCR with High Sensitivity
Source: PLoS Biol. 2008 Feb 26;6(2):e43. doi: 10.1371/journal.pbio.0060043 (PMC2253636; doi:10.1371/journal.pbio.0060043)
Supplement: Figure S9 — (A and B) Lipid bilayers containing 5 mol% DOGS-NTA-Ni were incubated with 1 μg/ml IEk-MCC. After washing, the bilayers were used to stimulate fura-2–pulsed AD10 T cells treated with 10 μM cytochalasin D for 1 h (B), or untreated T cells (A). Calcium flux was monitored using a 40× oil objective and displayed in pseudocolor. (C and D) The 12-well tissue culture plates were coated with 100 μg/ml streptavidin in PBS (pH 7.4) for 2 h at 37 °C. After washing, the plates were blocked with PBS (pH 7.4) containing 5 mg/ml BSA for 10 min and incubated with 1 μg/ml bio-IEk-MCC for 4 h at room temperature. After washing, the plate was used to stimulate fura-2–pulsed AD10 T cells treated with 10 μM cytochalasin D for 1 h (D), or untreated T cells (C). Calcium flux was monitored using a 60× water immersion objective and displayed in pseudocolor. (287 KB DOC) [file pbio.0060043.sg009.doc]

**Figure S9 (1.5 column-widths)**
