# Supplementary material for: Surface-Anchored Monomeric Agonist pMHCs Alone Trigger TCR with High Sensitivity
Source: PLoS Biol. 2008 Feb 26;6(2):e43. doi: 10.1371/journal.pbio.0060043 (PMC2253636; doi:10.1371/journal.pbio.0060043)
Supplement: Figure S10 — ELISA plates coated with 10 μg/ml IEk-MCC or IEk-99A were used to stimulate primed AND T cells in medium containing 20 μg/ml brefeldin A for 7 h. IL2 production was measured by intracellular staining and flow cytometry. (27 KB DOC) [file pbio.0060043.sg010.doc]

**Figure S10 (1 column-width)**
