# Supplementary material for: Surface-Anchored Monomeric Agonist pMHCs Alone Trigger TCR with High Sensitivity
Source: PLoS Biol. 2008 Feb 26;6(2):e43. doi: 10.1371/journal.pbio.0060043 (PMC2253636; doi:10.1371/journal.pbio.0060043)
Supplement: Text S1 — (26 KB DOC) [file pbio.0060043.sd001.doc]

**Text S1**

The difference in shape and range of the response curves to IEk on streptavidin versus ELISA plates may be caused by a combination of factors:

a) Different pMHC binding curves to ELISA plates versus streptavidin plates. Note that the dose responses in Figure 3C-3F of the manuscript were to pMHC coating concentration, rather than absolute pMHC surface density. Because of the totally different binding mechanisms, it is quite possible that pMHC binding to ELISA plates follows a different binding dose curve than to streptavidin plates. In support of this, about 1000 times higher concentration of agonist pMHC was needed to coat ELISA plates than streptavidin plates, in order to achieve similar levels of T cell activation. Also, from Figure S4 of the manuscript, it can be seen that the pMHC binding curve to ELISA plates is similar to the T cell response curve to pMHC coated ELISA plates.

b) Loss of function by pMHCs anchored on ELISA plates. In terms of the function of bound pMHCs, binding to ELISA plates should be much less efficient than to streptavidin plates. Because pMHCs were biotinylated at the membrane-proximal end of the molecules, all the molecules bound to streptavidin-coated plates should be in their native form and have the optimal orientation for effective TCR triggering. In contrast, non-specific binding of pMHCs to ELISA plates based on charge and hydrophobic interactions could lead to protein denaturation and incorrect binding orientations. Moreover, it is possible that such functional inefficiency of pMHC binding on ELISA plates increases as the protein coating concentration decreases. For instance, it is conceivable that a lone protein molecule is more likely to denature after contacting hydrophobic elements of the ELISA plate surface than a cluster of protein molecules, as more possible protein-plastic contact interfaces are available to a lone protein molecule. Such non-linear increases in functional pMHC binding inefficiency with decreasing pMHC coating concentrations may account for the shorter dynamic range observed with ELISA plates.

c) Different surface properties of streptavidin plates and ELISA plates in terms of promoting T cell adhesion. ELISA plates are not designed for cell culture purposes, thus T cells may adhere poorly to the surface. In contrast, streptavidin has been shown to contain the tripeptide sequence Arg–Tyr–Asp (RYD) that apparently mimics the Arg–Gly–Asp (RGD) binding sequence of fibronectin, a component of the extracellular matrix that specifically promotes cellular adhesion. Therefore, T cells may adhere much better to streptavidin plates. As shown in Figure 5, adhesion is required for TCR triggering by surface-anchored pMHCs. Better T cell adhesion to streptavidin plates may extend the dynamic range of T cell response to streptavidin plates in comparison to ELISA plates.
