# Supplementary material for: Surface-Anchored Monomeric Agonist pMHCs Alone Trigger TCR with High Sensitivity
Source: PLoS Biol. 2008 Feb 26;6(2):e43. doi: 10.1371/journal.pbio.0060043 (PMC2253636; doi:10.1371/journal.pbio.0060043)
Supplement: Text S2 — (38 KB DOC) [file pbio.0060043.sd002.doc]

**Text S2**

On plastic plates, 5% of T cells were activated when each T cell had contact with 0.83 agonist pMHCs on average. Random ligand distribution on the surface generates ‘count data’ and should follow a Poisson distribution. As shown in the table below, at this pMHC density, the percentages of T cells having contact with 3 or more agonist pMHCs is 5.64%, 4 or more is 1.15%, and 5 or more is 0.19%, and so on. Therefore, the 5% responding T cells might have contacted with as many as 3 agonist pMHCs.

| Number of pMHC per T cell | 0 | 1 | 2 | 3 | 4 | 5 | 6 |
| --- | --- | --- | --- | --- | --- | --- | --- |
| % of T cells | 42.32 | 36.39 | 15.65 | 4.49 | 0.96 | 0.17 | 0.02 |
| % T cells seeing **2 or more** pMHCs | | | 21.29 | | | | |
| % T cells seeing **3** **or more** pMHCs | | | | 5.64 | | | |
| % T cells seeing **4 or more** pMHCs | | | | | 1.15 | | |
| % T cells seeing **5 or more** pMHCs | | | | | | 0.19 | |
| % T cells seeing **6 or more** pMHCs | | | | | | | 0.02 |

On lipid bilayers, 5% of T cells were activated when each T cell had contact with 3 agonist pMHCs on average. As shown in the table below, the responding T cells might have contacted with as many as 7 agonist pMHCs.

| Number of pMHC per T cell | 0 | 1 | 2 | 3 | 4 | 5 | 6 | 7 | 8 |
| --- | --- | --- | --- | --- | --- | --- | --- | --- | --- |
| % of T cells | 5.0 | 14.9 | 22.4 | 22.4 | 16.8 | 10.1 | 5.0 | 2.2 | 0.8 |
| % T cells seeing **2 or more** pMHCs | | | 80.1 | | | | | | |
| % T cells seeing **3** **or more** pMHCs | | | | 57.7 | | | | | |
| % T cells seeing **4 or more** pMHCs | | | | | 35.3 | | | | |
| % T cells seeing **5 or more** pMHCs | | | | | | 18.5 | | | |
| % T cells seeing **6 or more** pMHCs | | | | | | | 8.4 | | |
| % T cells seeing **7 or more** pMHCs | | | | | | | | 3.4 | |
| % T cells seeing **8 or more** pMHCs | | | | | | | | | 1.2 |
